# Non-Canonical Helical Structure of Nucleic Acids Containing Base-Modified Nucleotides

**DOI:** 10.3390/ijms22179552

**Published:** 2021-09-02

**Authors:** Thananjeyan Balasubramaniyam, Kwnag-Im Oh, Ho-Seong Jin, Hye-Bin Ahn, Byeong-Seon Kim, Joon-Hwa Lee

**Affiliations:** 1 Department of Chemistry, Gyeongsang National University, Jinju 52828, Gyeongnam, Korea; dhanaj8@gmail.com (T.B.); orangekwang@gmail.com (K.-I.O.); dlxl0826@naver.com (H.-S.J.); ahb9004@naver.com (H.-B.A.); 2 The Research Institute of Natural Science, Gyeongsang National University, Jinju 52828, Gyeongnam, Korea; 3 Department of Chemistry Education, Gyeongsang National University, Jinju 52828, Gyeongnam, Korea

**Keywords:** C8-modification, C5-modification, methylation, bromination, arylation, Z-DNA, G-quadruplex, i-motif, triplex, non-canonical DNA

## Abstract

Chemically modified nucleobases are thought to be important for therapeutic purposes as well as diagnosing genetic diseases and have been widely involved in research fields such as molecular biology and biochemical studies. Many artificially modified nucleobases, such as methyl, halogen, and aryl modifications of purines at the C8 position and pyrimidines at the C5 position, are widely studied for their biological functions. DNA containing these modified nucleobases can form non-canonical helical structures such as Z-DNA, G-quadruplex, i-motif, and triplex. This review summarizes the synthesis of chemically modified nucleotides: (i) methylation, bromination, and arylation of purine at the C8 position and (ii) methylation, bromination, and arylation of pyrimidine at the C5 position. Additionally, we introduce the non-canonical structures of nucleic acids containing these modifications.

## 1. Introduction

**Figure 1:**
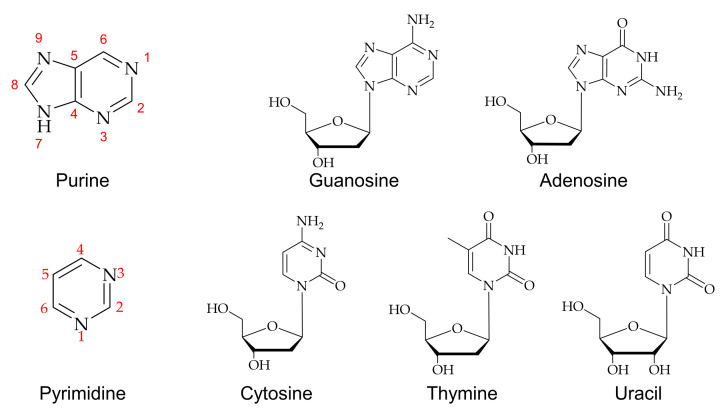
The organic structural formulas for the bases in nucleic acids, DNA and RNA. The numbering of elements in the molecules is indicated by red color.

In recent years, research on chemical alterations in nucleobases has increased as it is used for therapeutic purposes and the diagnosis of genetic diseases [1,2,3]. These nucleic acid bases can be naturally modified or chemically synthesized. In particular, many artificially modified nucleobases are widely used with methyl, halogen, and aryl groups of purine at the C8 position [3,4,5,6] and pyrimidine at the C5 position [7,8,9] (Figure 1). Despite the numerous studies conducted on this topic, most of the properties and functions of base-modified substituents have not yet been fully determined. There have been comprehensive studies and reviews investigating the synthetic methods used and structural studies of the artificial alteration of nucleobases.

The two ordinary purines—adenine and guanine—can undergo a conformational equilibrium between the anti and syn conformations. It is generally accepted that the sterically large substituent at the C8 position of the purine nucleotide shows a preferential structure for the *syn* glycoside conformation [6,10,11]. The typical conformation of the DNA duplex is that B-DNA forms an *anti*-conformation preferred by the DNA bases of A-T and G-C under physiological conditions [12]. Interestingly, solvents, agents, and chemical modifications promote non-B conformations of DNA, such as Z-DNA [13,14,15] and G-quadruplex (G4) [16,17,18]. This interesting fact about non-canonical structural sequences is correlated with disease-inducing genes and plays a vital role in biological functions. For instance, Z-DNA-forming sequences are identified near transcription initiation sites [19,20].

Base modifications play a pivotal role in forming alternative structures from DNA and RNA. The modifications at the C8 position of purines are used to study Z-DNA and G-quadruplex as potent secondary structure inducers [21]. Interestingly, chemical modifications of DNA base or naturally modified DNA lead to other alternative structures in DNA such as H-DNA and triplex helix formation [22]. On the other hand, the C8- modifications of RNA are supported to form Z-RNA and RNA-quadruplex structures from locally known RNA structures [23]. Since C5 methylation of tRNA sequence changes base pairing to Levitt base pairs instead of Watson–Crick base pairs, the methylated guanosine at the C5 position interferes with the role of tRNA and forms RNA duplexes.

Despite the numerous studies conducted in this field, the roles and conformational properties of non-canonical DNA structures, including *syn* conformations, are not yet fully understood. Typically, non-canonical structures form under certain conditions: (i) higher-salt conditions favor Z-DNA; (ii) guanine-rich sequences form the tetraplex of G4. Thus, it is necessary to mimic or induce alternative conditions for further investigation. This review discusses the synthesis of chemically modified nucleotides and oligo-nucleotides containing modified bases and their effects on the non-canonical structures [24].

## 2. Synthesis of C8-Modified Purine

Several purine modifications have been observed in DNA. Most purine modifications are correlated with DNA mutations, such as oxidation, methylation, and nitration. The purine modification mainly focuses on the C8 position. Over the past 40 years, numerous modifications have been synthesized, such as methylated purine, brominated purine, and arylated purines at the C8 position [25,26,27,28,29]. These modified purines show potential for biological analysis and the investigation of the secondary structures of DNA [30,31,32]. This section describes various alterations of guanosine, including some introductions of adenosine.

### 2.1. Synthesis of C8-Methylpurine

DNA methylation is the addition of a methyl group to a nucleic acid base, and it will occur naturally in the cell or the methylation caused by endogenous and exogenous electrophiles [33]. A single methylation can change the role of a particular DNA segment without altering other sequences. Moreover, methylation is an essential mechanism for normal development and has been implicated in biological activities such as the repression of gene transcription, genomic imprinting, and carcinogenesis [33,34,35]. The chemically modified purine at the C8 position possesses therapeutics activity like 8-methyl guanosine induced to release the tumor necrosis factor α (TNF-α) [36]. The various chemicals of methylated purine bases have been used by different methods and positions of methylation in DNA [37,38,39]. Several nucleophilic sites of DNA, such as guanine at the N3 and C8 positions and adenine at the N1, N3, and C8 positions, have been used for producing various types of methyl purines [37].

**Figure 2:**
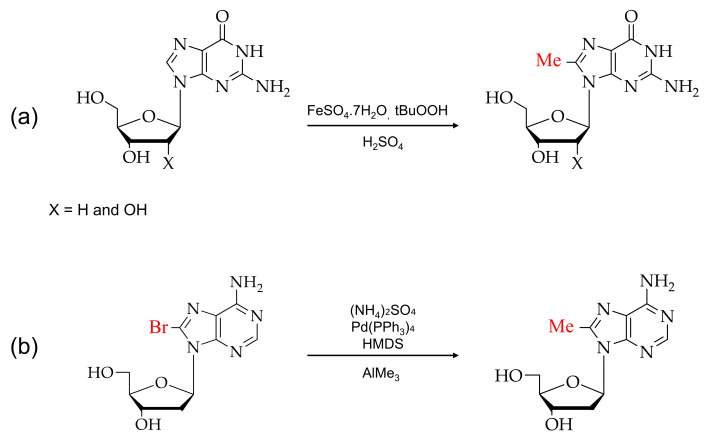
Methylations of purines: (**a**) 8-methylguanosines and (**b**) 8-methyladenosines. Bromide and methyl groups are highlighted.

An efficient method for C8-methylation in guanine, adenine, hypoxanthine, and its derivatives is a metal ion mediated radical reaction that generates the radical in an acidic medium to form methylated nucleosides [39,40,41]. A free radical methylation method was introduced using *t*-butyl hydroperoxide (TBHP) as a source of methylation catalyzed by ferrous ions [42]. Methylation of guanosine and its derivatives has been found to give good yields in acidic conditions due to the influence of C8 carbon on the electronic properties of 2,6-substituents on a purine nucleoside ring [39,42,43,44,45,46]. Figure 2a shows a general methylation reaction scheme for guanosine. Naturally formed methylated nucleic acid derivatives were investigated using deuterium labeling with methylated nucleoside [33]. An electron-impact fragmentation pattern showed that guanine is more advantageous for obtaining structural information from mass spectroscopy than those of adenine or hypoxanthine due to high reactivity. Similarly, a detailed analysis of the C8-methyl substitution reaction of guanine reported by Eistetter and coworkers concluded that N-7 and N-9 substitution also occur during the methylation isolated products from the reaction [47].

8-methylated adenosine was successfully formed by a palladium-catalyzed cross-coupling reaction of 8-bromoadenosine, as shown in Figure 2b [46,48,49]. This cross-coupling reaction made it applicable to incorporate an alkyl group into various nucleosides for further biochemical studies.

### 2.2. Synthesis of C8-Bromopurine

The brominated purine nucleosides at the C8 position are capable of inducing high-level proliferation of lymphocytes in the presence or absence of a serum condition [50]. 8-bromoadenosine acts as a very selective ligand for the A3 adenosine receptor subtype, behaving as an adenosine antagonist [51]. Brominated adenosine derivatives at the C8 position induce a delayed chain termination in vitro and have been proven to moderate HIV activity in cell culture [52,53].

**Figure 3:**
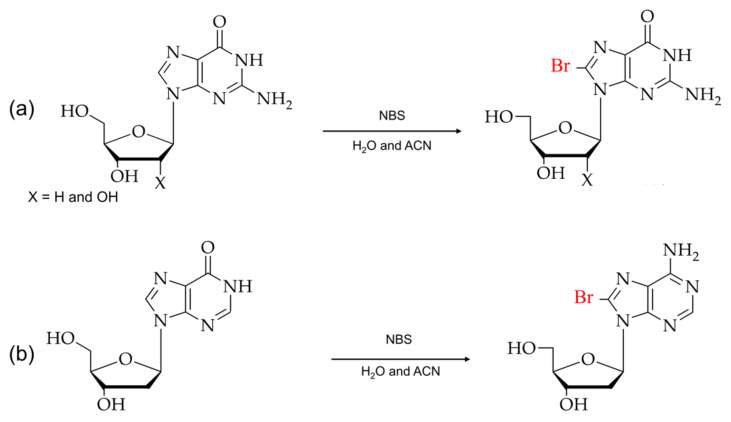
Typical brominations of purines: (**a**) 8-bromoguanosines and (**b**) 8-bromoadenosines with N-bromosuccinimide (NBS).

Over the past few years, various reagents have been employed for the direct bromination or insertion of bromine at the C8 position of purine analogs. There are two types of modifications: the direct bromination of the C8 position of purine monomer and post-DNA synthesis modification [54,55]. Figure 3 shows the reaction scheme of guanosine and adenosine with N-bromosuccinamide (NBS) for direct bromination at the C8 position [56,57,58].

### 2.3. Synthesis of C8-Arylpurine

The formation of new carbon–carbon bonds is particularly important in nucleoside modifications caused by transition metal-catalyzed cross-coupling reactions. These cross-coupling reactions are important for the synthesis of an agro-chemical, organic compound, and pharmaceutical. Cross-coupling reactions have been used in the synthesis of biaryl and heteroaryl compounds, including purines arylated by nucleophilic substitution or metal-mediated reactions [59,60,61,62]. 8-arylated purines have been used as the antivirus activators, anti-Parkinson agents, and adenosine receptor antagonists [63,64]. In addition, 8-arylated purines are widely used as a marker in the structural analysis of nucleic acids, therapeutic agents, and epigenetics [65,66,67].

**Figure 4:**
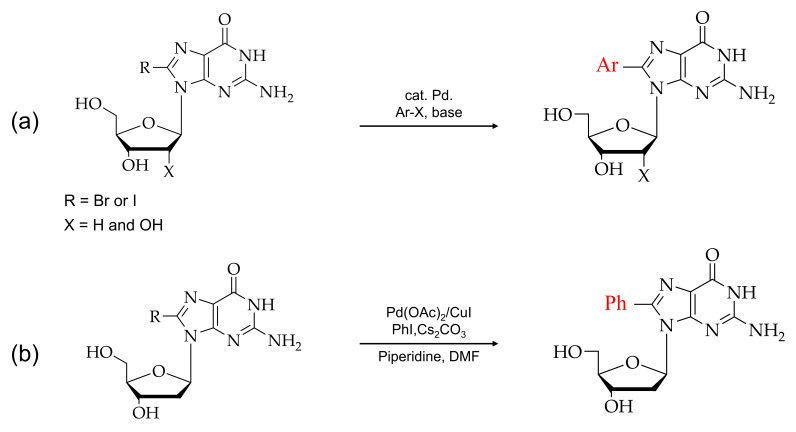
(**a**) Typical synthesis of 8-arylated guanosine. (**b**) Scheme for direct arylated guanosine derivatives at the C8 position.

As shown in Figure 4, the direct arylation at the C8 position in purines is caused by the metal-mediated cross-coupling reactions, which use unprotected halogenated nucleosides as a starting material. The cross-coupling reactions in water-soluble reagents are cost-effective and eliminate the additional steps, such as deprotecting the protective functional groups in nucleosides [68,69,70,71].

Furthermore, the cross-coupling reaction conditions and yields of the product depend on various water-soluble phosphine ligands. For example, tripheylphosphine trisulfonate (TPPTS) and Tris(2,4-dimethyl-5-sulfophenyl) phosphine trisodium salt (TXPTS) are more effective phosphine ligands. Comparing ligands activities, TXPTS was reported to have a higher-level conversion rate of product (~99%) in a 17-hrs conservation time scale than TPPTS (70%). Despite the C8-arylated nucleotide conversion rate depending on halogenated nucleobases, 8-BrdG has a lower conversion rate of development than 8-BrdA. There are also other factors involved in the conversion of C8-aryl nucleotides. For example, at low pH, the N-7 coordination of guanine with palladium (II) is favored, whereas, at the higher pH, the N-1 coordination is preferred.

The recent development of the direct arylation of DNA sequences has some advantages over compared to the arylation of monomers. This method is known as post-synthesis modification. Site-specific modification is carried out by replacing halogenation, such as Br or I with desired aryl groups [72].

Moreover, the post-synthesis of DNA modification in a guanine-base methodology has been developed [72]. Direct C-H arylation was performed using the cross-coupling, which was reported as the first-time feasibility of a cross-coupling reaction for the synthesis of C8-arylated guanine-modified oligonucleotides. Natural fungal carcinogens were used as substrates for cross-coupling reactions. Excellent yields up to 15 mer can be modified with this protocol.

**Figure 5:**
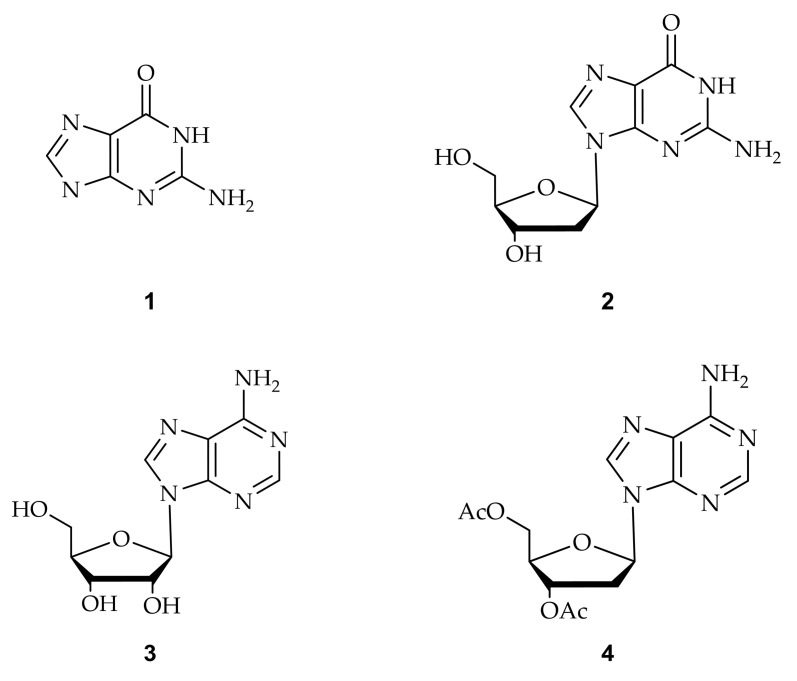
Purines substrates (**1**: guanine, **2**: 2’-deoxy guanosine, **3**: 2’-ribose adenosine, and **4**: 3’,5’-di-O-acetyl-2’-deoxy adenosine) are involved in the acylation employed by palladium catalyst.

Another exciting C8 modification is the addition of an oxidative group at the C8 position of purine and the synthesis of the 8-oxo dG [73]. The 8-oxo purines are also used to study non-canonical structures, such as C8-aryl purines. The sequence of an oxidative form of the 8-oxo dG is recognized by formamidopyrimidine-DNA (Fpg protein) [74]. Various research groups recently developed efficient methods for synthesizing C8-aryl purines by direct arylation [62,75,76,77,78]. Table 1 summarizes the different functionalized C8-aryl purines syntheses from various purine derivatives (Figure 5).

## 3. Non-Canonical Structure Containing C8-Modified Guanosine

Modified nucleosides at the C8 position have been incorporated into oligonucleotides to unveil the structural characteristics and biological significances [5,7,21,79]. This section reviews the major contributions of nucleoside alterations to the investigation of Z-DNA and G-quadruplexes.

### 3.1. Z-DNA

Z-DNA forms a left-handed helix where the base pair is located almost perpendicular to the phosphate backbone, while B-DNA represents a right-handed helical structure. Significant factors influencing the B-Z transition have been identified, such as alternating (CG)_n_ sequences, high salt concentrations, multivalent cations or polycations, and Z binding proteins [14,80,81,82,83]. In addition, steric effects are also important. Since bulky substituents at the C8 position induce a *syn* conformation with a Z-DNA structure, several base modifications at the C8 position, including 8-methylation, -bromination, and -arylation, have been studied in connection with the B-Z transition [5,6]. Oligomers containing chemically modified nucleotides can remarkably stabilize the Z-form of DNA. These modified oligomers were used to investigate the Z-form binding proteins, the most extensively favorable sequence for Z-form study used as CG repeat sequences. Still, some recent studies show that the d(TA)n sequence can be without purine-pyrimidine repeats and also form Z-DNA and interact with ADAR1 protein. The Zα domain of the ADAR1 protein interacts effectively [84]. The interdependence between the proportion of B-DNA and the Z-DNA conformation of the modified DNA and salt concentrations is shown in Table 2, which presents the preferential equilibrium.

#### 3.1.1. C8-Methylation

**Figure 6:**
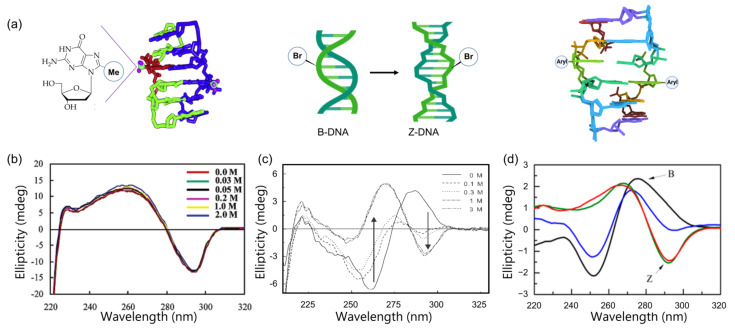
Graphical representations and CD spectra of the preferential structure of modified DNA. (**a**) DNA structure including purines modified with methylation (left), bromination (middle), and arylation (right); (**b**) CD spectra of 8-methyl ribo-guanosine incorporating oligomer stabilized the Z-DNA at low salt concentrations; (**c**) B-Z transition monitored by CD spectra. 8-Bromo-2′-deoxyguanosine containing an octamer sequence (d(CGT^Br^GCACG)_2_) dramatically stabilized the Z-form in low-salt physiological conditions; (**d**) CD spectra of the natural sequence and modified hairpin sequence in salt physiological condition from Ref. [89]. Arrows indicate the signature band of Z-DNA. Copyright (2003), (2004), and (2014), American Chemical Society.

The methylation of guanosine at the C8 position remarkably stabilizes the Z-conformation of oligonucleotides under physiological salt conditions [79,85,86]. The characterization of structural preferences was determined by CD spectra and the NMR of oligonucleotides incorporating 8-methylguanosine (Figure 6a,b). Figure 6 represents the signature bands of the CD spectral pattern for Z-DNA at 265 and 290 nm with positive and negative signs, respectively. Sugiyama et al. investigated the thermodynamic parameters of DNA B-Z transition for oligonucleotides containing 8-methylguanosine, and reported a reduction in entropic penalty by enthalpic gain during a Z-DNA formation [85]. Later, preferential structures of Z-DNA and RNA were investigated by CD spectra, and fluorescence detection was carried out by an electrophoretic mobility shift assay [86,87,88].

**Table 2:** The midpoint of NaCl concentrations for the B to Z transition of DNA in various base-modified monomers incorporating d(CGCGCG)_2_ and poly(dG-dC).

Entry	Sequence	Modification	Midpoint of (B–Z Transition) NaCl Concentration (mM)	Reference
1	d(CGCm^8^rGCG)_2_	m^8^rG	0	[79]
2	d(CGC**^F^G**CG)_2_	**^F^G**	20	[88]
3	d(CGCm^8^mGCG)_2_	m^8^mG	0	[86]
4	d(CGCm^8^GCG)_2_	m^8^G	30	[90]
5	Poly(dG-dC)	Br-Poly(dG-dC)	1000	[91]

#### 3.1.2. C8-Bromination

Brominated nucleobases, including poly(dG-dC), promote the stabilization of Z-DNA in low-salt physiological conditions [92]. As a result of bromination, 45% and 20% of guanine and cytosine, respectively, are brominated. The midpoint of B-Z transition is 0.15 M for brominated poly(dG-dC), which shows similar structural characteristics to those of unmodified poly(dG-dC) at 4M NaCl (Figure 6c) [91]. The shifts in equilibrium between B and Z conformations occur due to the steric hindrance of bromo-substitution at the C8 position. Moreover, the substitution of the bromo group interacts with the phosphate backbone of the DNA to form a *syn* conformation [93,94,95].

#### 3.1.3. C8-Arylation

**Figure 7:**
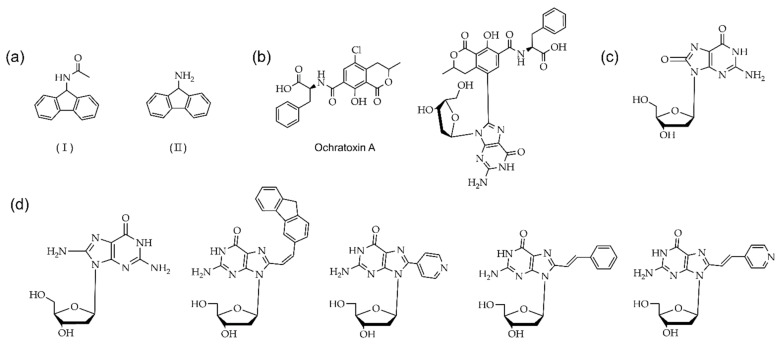
Carcinogenic adducts guanosine derivatives at the C8 position: (**a**) (I) The AAF and (II) AF; (**b**) Ochratoxin A and 8-OTA dG; (**c**) 8-oxo G; (**d**) 8-arylated compound at the C8 position, 8-aminoguanine (n8G), 8-(2-pyridyl)guanine (2PyG), 8-(2-phenylethenyl)-dG and 8-[2-(pyrid-4-yl)- ethenyl]-dG and 8-fluorenylvinyl-dG (Fv8G), respectively.

Arylation at the C8 position of guanosine is an important modification for the investigation of non-canonical structures, and these modified nucleosides also act as an internal probe. The carcinogenic adducts used for the substitution of guanosine at the C8 position have a significant influence on Z-DNA formation (Figure 7) [28,89,96,97]. The acetylaminofluorene (AAF) and aminofluorene (AF) adducts substituted into the C8 position of guanosine incorporated in poly(dG-dC)-AAF can adopt Z-form DNA without the presence of alcohol [98]. Recently, it was reported that guanosine inserted by ochratoxin A (OTA) into the C8 position can also adapt Z-form conformations, such as AAF and AF [99,100,101]. The 8-aryl-dG also destabilizes the B-DNA conformation and favors the Z-DNA conformation, since the steric factor helps glycosidic bond rotation to form *syn* conformations [32]. Those 8-arylated compounds exhibit a midpoint of B-Z transition at 0.6–0.8 M NaCl solution (Figure 7d) [89]. The synthesis of trifluoromethyl adduct involves guanine and incorporates oligonucleotide for the examination of the ^19^F NMR. The results show that 8-trifluoromethyl 2′-deoxyguanosine strongly supports the Z-DNA conformation in in vivo conditions [88] and C8-arylated guanosine is recognized by repair proteins, Rad14 and XPA [102].

### 3.2. G-Quadruplex

G4 is a four-stranded helical structure containing a G-tetrad stack, which is the result of the planar association of four guanines through Hoogsteen hydrogen bonds forming a cyclic square of G4 [16]. G4 was observed early in the self-assembly of guanylic acid and supports the elucidation of G-tetra-forming sequences. In the formation of G-tetra or G4, the guanine combines with three or more of each other to form a planar arrangement stabilized by positively charged ions and lone pair electrons from O6 of guanines. The monovalent ions, M+, are well known as the stabilizers of G4 among cations, and K+ is the most favorable cation [103,104,105,106,107]. Modified guanosines with relatively small substituents at the C8 position, such as bromine, methyl, and oxymethyl, have been used to investigate the structures of G4s [108,109,110]. There is an excellent review that interprets the CD spectra of G4 structures formed by the incorporation of 8-substituted guanosines [31].

#### 3.2.1. C8-Methylation

**Figure 8:**
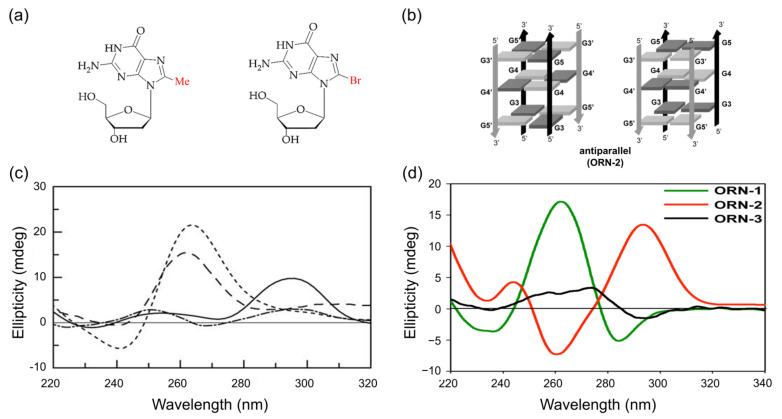
(**a**) Graphical representation of 8-methylguanosine and 8-arylated guanosine; (**b**) stabilized anti-parallel structures of G-quadruplexes in K+ solution; (**c**) CD spectra of G-quadruplex sequence, which contains 8-methyl guanosine: ([d(TGGGT)]_2_) at 20 °C (dotted), Q1 at 20 °C (solid), Q2 at 20 °C (dashed), and Q3 at 5 °C (dashed dot); (**d**) CD spectra of ORN-1 (UA(8^Br^rG)GGU, red solid line, parallel G4 structure), ORN2 (UAG(8^Br^rG)GU, green solid line, anti-parallel G4 structure), and ORN3 (UAGG(8^Br^rG)U, black solid line, single-strand structure). Copyright (2017) Springer Nature, and (2005) Oxford University Press.

The methyl-substituted guanosine at the C8 position (8-Me-dG) is also one of the stabilizers of G4 structures [111]. The patterns of CD spectra, along with the directions of G-tetra, are shown in Figure 8. For example, the incorporation of 8-Me-dG into (TGGGT)4 sequences at the second position gave an anti-parallel G4 in K+ ion solution, showing its CD spectra profile, which has a positive peak at 290 nm. In contrast, inserting 8-Me-dG at the third position caused a strong positive peak at 260 and a minor negative peak at 240 nm (Figure 8c) [111,112,113].

The biological significance of sequences containing 8-Me-dG has been investigated by the retinoblastoma gene, which acts as a tumor suppressor by interfering with the cell cycle. 8-Me-dG incorporates 18mer sequences that switched conformations between anti-parallel G4 with G-tetrads at neutral pH [37].

#### 3.2.2. C8-Bromination

The bromination of guanine has been shown to have synthetic and biological significance, along with an advantageous glycosidic bond rotation *syn* conformation. [48,85,113,114,115]. The incorporation of brominated guanosine (8-Br-dG) into oligonucleotides increases the thermal stability along with the number of substitutions. The thermal stability of oligonucleotide increases by 5–6 °C, while the other four additions of 8-Br-dG into oligonucleotides reduce the stability by 3–6 °C [116]. Different topological structures containing 8-Br-dG in different locations were characterized by the CD spectra. An anti-parallel G4 shows a positive cotton effect at 295nm and a negative peak near 260 nm [108,117,118,119].

#### 3.2.3. Other Modifications at the C8 Position

The 8-oxoguanine (O^8^G) substitution preferred the form of a *syn* conformation (Figure 7d). The substitution at the 5′ position led to the formation of tetra molecular G4 [120]. The effect of O^8^G on the activity of the telomerase enzyme was investigated in the electrochemical measurements [121]. The substitution of O^8^G at 8 and 14 positions in G4 contributes to decreasing the telomerase activity, but the substitution of the GGG triplet at 9 and 15 positions led to an increase in the enzymatic activity.

8-amino-2′-deoxyguanosine (8AG) is one of the mutagenic intermediates observed in cellular DNA. 8AG (Figure 7d) can destabilize parallel, anti-parallel, and triplex formations due to the increase in the free energy of each substation of 1–4 kcal/mol [122]. The G4 structure is destabilized at 7 °C due to the incorporation of 8AG into the 15 mer DNA, while the unmodified 15 mer forms an anti-parallel G4 structure. The CD spectra profile data show that the 8-amino-2′-deoxyguanosine (8AG) incorporated a GGTTGGTGTGGTTGG sequence, different from the unmodified 15 mer structures.

Internal fluorescent probes have more advantages in terms of detecting the structural properties of G4. 8-(2-pyridyl) guanine (2PyG) is an internal fluorescent compound, as the coordination between G4 and cations induces efficient DNA-to-probe energy transfer [114]. The coordination of cations at the O6 position produced a strong energy transfer due to the smaller distance between the cation and 2PyG at the turn site. Two fluorescent probes, 8-(2-phenylethenyl)-dG and 8-[2-(pyrid-4-yl)-ethenyl]-dG, show a high structural selectivity [123,124]. Forming a G4 structure results in a stronger fluorescence signal than single-strand or duplex because these internal probes detect the characteristics of secondary structures and spectroscopic properties. Interestingly, the structural changes in the 8-fluorenylvinyl-dG (Fv8G) are able to control the G4 formation [125]. This photo-switchable G4 formation can be applied to the biological events involving G4s.

## 4. Synthesis of C5-Modified Pyrimidine

**Figure 9:**
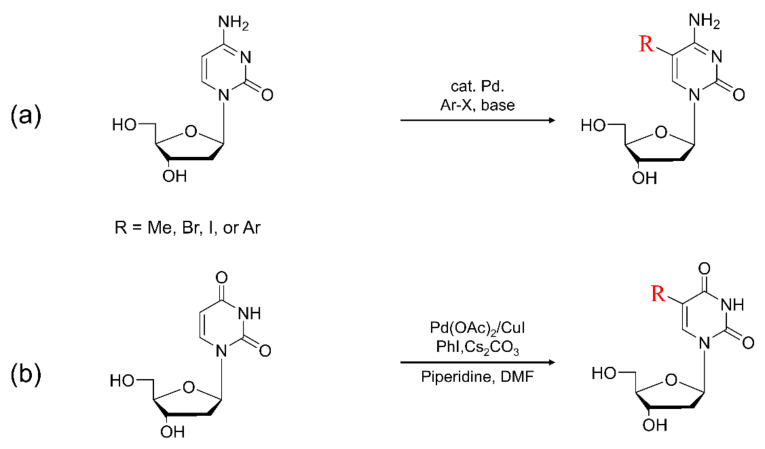
(**a**) Typical synthesis of 5-arylated cytosine. (**b**) Scheme for direct arylated uracil derivatives at the C5 position.

Several pyrimidine modifications have been observed in DNA. Most of the changes are correlated with DNA mutation, such as oxidation, methylation, and nitration. The purine modification mainly focuses on the C5 position. Numerous modifications at the C5 position containing purine have been synthesized in the past four decades, such as methylated pyrimidines, brominated pyrimidines, and arylated pyrimidines (Figure 9). These modified purines represent potent candidates for biological analysis and are used to investigate secondary structures of DNA [44,126,127,128,129].

### 4.1. Synthesis of C5-Methylpyrimidine

Cytosine is commonly methylated at the C5 position, as it is the most abundant endogenous modification of DNA with approximately 5% of all cytosine bases carrying a C5 methyl group [130]. 5-methylcytosine plays a crucial role in the tissue-specific gene expression pattern, the inactivation of X-chromosome activity, and genomic imprinting [131,132,133]. The 5-methyl uracil used for the photochemistry as the probe has a potency of antimicrobial activity [134,135,136,137,138]. Reactions of 5-substitution for pyrimidine have been developed based on the C-H activation, palladium coupling reaction, and the addition of formaldehyde [8,139,140]. In addition, efficient methodologies have been reported for the synthesis of methylated pyrimidines [141,142,143,144,145]. Methylated and hydroxymethylated cytosines are recognized by restriction endonuclease enzyme (LpnPI), zinc-finger protein Kaiso and SUVH5 [146].

### 4.2. Synthesis of C5-Bromopyrimidine

The C5-bromination of pyrimidine has significant antimicrobial properties that can be used for therapeutic purposes [50]. For instance, it was recently reported that the 5-bromoethylnyluridine possesses anti-HCV properties [147]. 5-bromopyrimidines are used for the structural elucidation of mismatched base pairs with normal base pairs to explain mutagenic pathways and properties. C5-bromination, used for the preparation of antisense, also utilizes for the radioactive labeling, stabilization for a triplex, and investigation of endonucleases activity [148,149,150,151]. Various 5-halopyrimidines and their derivatives have biological effects such as antibacterial activity, oncology therapeutics, and are involved in the investigation the nucleic acid damage and metabolism [50]. Halogenated pyrimidines are used for labeling. For example, 5-BrU is labeled with RNA during the RNA synthesis process [152] and 5-BrU contains DNA-RNA hybrid duplex sequences with polypurine RNA sequences. 5-BrU was also used to study the A-form structure of DNA-RNA hybrid duplex by X-ray crystal structure [153]. C5-bromination or iodination has been achieved using halogenation agents, such as *N*-iodosuccinimide, nitric acid, or *N*-Bromosuccinimide [49,154,155,156,157].

### 4.3. Synthesis of C5-Arylpyrimidine

Pyrimidine nucleosides substituted at the C5 position have a high potential for antiviral, antibacterial, and anti-fungal activity [155,158,159,160]. Pd-catalyzed cross-coupling reactions have been extensively developed that possess over the past 40 years and have become one of the important methodologies for arylation at the C5 position of nucleosides [68,139,161,162,163]. For example, the arylation at the C5 position of the uracil ring has been accomplished by various coupling reactions, such as the Suzuki coupling reaction [68,164,165,166,167]. Despite the critical role of the arylation at the C5 position in pyrimidines, the direct C-H arylation of pyrimidine nucleoside bases is nowadays limited to uracil nucleoside bases [25,160,168,169,170]. This is because the C–H bond functionalization of pyrimidine is difficult for controlling the regioselectivity due to the acidic characteristics of C5 and C6 protons. More developments of C–H bond functionalization to pyrimidine analogues have been performed. For effective reactions, for the regioselective C-H arylation reactions, various agents have been used such as the activated palladium precursor complexes, carbonate, and acetate [171,172,173].

## 5. Non-Canonical Structure Containing C5-Modified Pyrimidine

**Figure 10:**
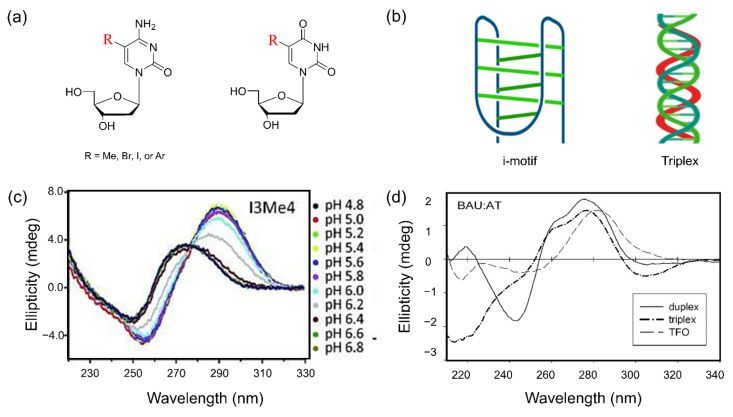
(**a**) Graphical representation of modified cytosine and uracil at the C5 position; (**b**) structures of i-motif and Triplex; (**c**) CD spectra of i-motif-containing modified cytosine at different pH indicated by different colors, from ref. [203]; (**d**) comparison of CD spectra between duplex (solid), triplex (dashed dot), and triplex-forming oligonucleotides (TFO, dashed) containing modified sequence at pH 6 from ref. [212]. Copyright (2015) John Wiley and Sons, Inc., and (2004) Oxford University.

Chemical modifications of nucleoside at the C5 position can provide more stability to non-canonical structures of oligonucleotides. These systems have been developed to understand their structure, folding properties, and biological importance. Here, we highlight the major contributions of nucleoside modifications to the study of Z-DNA, G-quadruplexes, i-motifs, and triple helices. The characteristics of structural preferences in non-canonical structures containing modified bases were determined by spectroscopic profiles (Figure 10).

### 5.1. Z-DNA

Several studies have shown that chemically modified nucleosides enable oligonucleotides to stabilize Z-form rather than B-form [174]. For instance, the methylation of cytosine is able to induce the B-Z transition of DNA in synthetic d(Gm^5^C)_n_ polynucleotides [175,176,177]. Other modifications of the d(GpC) also stabilize the Z-form of DNA by substitutions, such as iodine, bromine, and aza, at the C5 position [178]. More details are discussed in this section.

#### 5.1.1. C5-Methylation

The most predominant modification associate with gene silencing is methylation of cytosine at the C5 position. Oxidation of pyrimidine naturally occurred in a process catalyzed by translocation (TET) family proteins. The common oxidative methylation is methylation at the C5 position of pyrimidines. Several C5-modified cytosines such as 5-formylcytosine (5fC), 5-hydroxymethylcytosine (5hmC), and 5-carboxylcytosine (5caC) can be involved in C5-methyl cytosines mediate gene expression [179,180,181,182]. The presence or absence of methylation in the CG sequence is in favor of forming Z-DNA. In comparison with unmodified poly(CG), poly(m5CG) sequences show a significant potential to form Z-DNA, in particular, under the divalent cation such as Mg^2+^, which was used for prior Z-DNA studies [175]. Spectroscopic experimental results, along with calculations, support that van der Waal interaction with hydrophobic elements on the molecular surface may contribute to forming Z-DNA [13,183,184,185]. Moreover, sugar modification both with unmodified and modified bases of d(CG)_n_ tends to form Z-conformation [174,186]. Simulation trajectories can provide the molecular characteristics of the role of methylation in CG repeated units [187]. Although AT base pairs form fewer Z-conformation than CG base pairs, oligonucleotides containing AT base pairs can also form Z-DNA with C5-methylcytosine [177]. It has been suggested that disordered hydrating water molecules may contribute to the position reducing the relative stability of the Z-form to CG base pair.

#### 5.1.2. C5-Bromination

Bromination of cytosine at the C5 position in CG sequences can stabilize Z-conformation in common with methylation at the C5 position [91,93,188]. In high-concentration solutions, brominated cytosine produces Z-DNA conformations similar to unmodified DNA [91,189,190]. In addition, both DNA and RNA containing bromination at C5 can stabilize Z-conformation at low salt physiological conditions [191]. The structure of Z-RNA allows hydroxyl groups at the sugar backbone of guanosine to be exposed to the surface of the helix, which can involve in the recognition of protein binding.

### 5.2. G-Quadruplex

C5-methylation of cytosine can induce conformational changes of the G-quadruplex structure. Whereas the wild-type G-quadruplex sequence exhibits a mixture of parallel and anti-parallel structure [192], the corresponding sequence containing 5-methyl cytosine showed only parallel G-quadruplex structure, which typically shows positive and negative CD profiles at 265 and 240 nm, respectively (Figure 7b,c) [193]. It was demonstrated that the methylated cytosine involves a formation of G-quadruplex that interferes CCCTC-binding factor along with the flipping process from hairpin to quadruplex [194].

The replacement of 5-halogen modified deoxyuridine (dU) instead of T in the DNA sequence also affected the G-quadruplex conformation. The htel-22mer, AG_3_(TTAG_3_)_3_, and [G_4_T_4_G_4_]_2_ sequences containing the 5-iodo dU (5I-dU) instead of T prefer to form an anti-parallel G-quadruplex structure under Na^+^ condition rather than a mixture of G-quadruplex structures [195]. Interestingly, under K+ conditions, these 5-halogenated dU sequences folded different loop structures to G-quadruplex structures of the same modified DNA under Na^+^ conditions [196].

### 5.3. i-Motif Structure

The i-motif structure is one of the non-canonical structures of nucleic acids that have two parallel duplexes held together by hemiprotonated cytidine and protonated cytidine (C:C+) base-pairs intercalated in an antiparallel orientation (Figure 10b) [197,198]. The i-motif requires the protonated bases and thus is more stabilized under an acidic pH condition [199]. It was reported that the chemically modified cytosine exhibits different effects on the formation of i-motif structure under physiological conditions [200,201,202]. The halogenation of dC at the C5 position leads to the acceleration of the folding kinetics in the i-motif structure [203]. In addition, the incorporation of a methyl group at the C5 position of cytosine into the i-motif sequence increases the stability and thus changes pH dependence [203,204,205]. CD spectra for i-motif modified by methyl group show variation of profiles due to change of folded fraction at different pH (Figure 10c).

The interaction between hemi protonated C:C+ base pairs that form hydrogen bonds of i-motif in oligonucleotides have been investigated by various approaches such as base-pair opening kinetics, FRET experiments, and the base-pairing energy (BPE) calculation [206,207,208,209,210]. It has been demonstrated by NMR results that motion consists of a cooperative switch between two conformations of loop1 and loop3 of i-motif containing modified sequence [209]. Moreover, the BPE of the C:C+ base pair for methylated cytosine at the C5 position is much greater than the canonical Watson–Crick base pair [210]. In addition, the chemically modified nucleobases are used to form an i-motif structure of DNA under the neutral physiological condition [211].

### 5.4. Triple Helix

A triplex structure of nucleic acids is formed by additional Hoogsteen pairing of pyrimidine or purine bases, which occupy the major groove of the double helix, with purines of the Watson–Crick base-pairs [213]. Intermolecular DNA triplexes are formed by the interaction of triplex-forming oligonucleotides (TFOs) with target unique sequences by forming triplex following hydrogen bonding interactions between TFOs and the oligopurine strand of the duplex (Figure 10b). The TFOs containing the chemically modified nucleotide, particularly modified pyrimidine, significantly stabilize the formation of triplex structure than unmodified TFOs [214,215,216]. The structural property of triplex shows significantly different CD profile to CD spectral patterns of DNA duplex or TFO (Figure 10c). In addition, various analogs of modified uracil at the C5 position have been explored for the investigation of sequence selectivity and affinity of TFO [217]. TFO incorporated with C5-alkynyl functionalized LNA monomers dramatically stabilizes triplex formation and increases thermal stability [218]. The 5-(1-propynyl)-2′-deoxyuridine (pdU)-modified TFO, which targeted the A-rich site, formed the stable triplex structure, and required a lower concentration of Mg^2+^ cation to form triplex compared to unmodified TFOs. Interestingly, the pdU contains the TFOs more effectively than T-containing TFOs to direct the DNA. There are comprehensive reviews of the structural studies of the triplex based on chemical modifications [5,219,220].

## 6. Conclusions

Knowing the chemical alterations of purine nucleosides is important to better understand the structure and function of non-canonical DNA. Nucleoside modifications, such as methylation, bromination, and arylation, especially at the C8 position of purine and the C5 position of pyrimidine, are powerful tools not only to analyze the sequence-dependent structure of DNA, but also to investigate its roles in biological processes. For example, methylated nucleosides are used to investigate epigenetic regulation of cellular differentiation mechanisms.

This paper provides an overview of how the structure of non-canonical DNA can be influenced by DNA alterations at the C8 and C5 positions of nucleosides. Modified bases contain DNA sequence stability that differs from natural sequences. Most of the modifications either destabilize the local DNA structure or induce the formation of alternative secondary structures [221]. The recent studies of dsDNA or siRNA containing modified purines or uridines show that more modified bases actively alter genomic stability [222]. We predict that the effort to discover the underlying principles behind the link between local structure and base modifications will provide the key to elucidating the mechanism of how modifications affect genetic stability.

The developments of a more efficient methodology for the direct chemical modification at the specific position have been applied using brominating agents or Pd catalysis synthesis protocols. The field of research on pyrimidine arylation is narrow, as most of the pyrimidine arylation mainly focuses on uracil and its derivatives that have therapeutical roles. Further developments of the chemically synthetic methods and the characterization of the structural properties of modified DNA will make it possible to unveil the function of non-canonical DNA.

## Figures and Tables

**Table 1:** Palladium (Pd) mediated the direct arylation at the C8 position of various purine derivatives. The structures of substrates are described in Figure 5.

Substrate	Entry	Base/Ligand and Solvent	Temp (°C)	Aryl	Yield (%)	Reference
1	1	NaCO_3_/TPPTS DMSO	90 °C	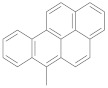	91%	[75]
2	2	NaCO_3_/TPPTS H2O	80 °C	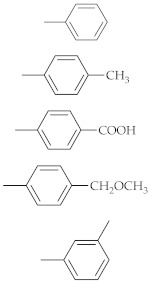	85%	[76]
	3	NaCO_3_/TPPTS H_2_O	80 °C		67%	
	4	NaCO_3_/TPPTS H_2_O	80 °C		83%	
	5	NaCO_3_/TPPTS H_2_O	80 °C		83%	
	6	NaCO_3_/TPPTS H_2_O	80 °C		72%	
3	7	CuI/piperidine DMF	150 °C	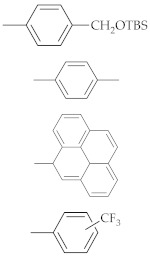	55%	[62]
	8	CuI/piperidine DMF	150 °C		68%	
	9	CuI/piperidine DMF	150 °C		55%	
	10	Cs_2_CO_3_/CuI DMF	120 °C		66%	[77]
4	11	CuI/PPh_3_ THF	90 °C	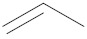	95%	[78]

Substrates are described in Figure 5.
